# Synthesis of 5′-GalNAc-Conjugated Oligonucleotides: A Comparison of Solid and Solution-Phase Conjugation Strategies

**DOI:** 10.3390/molecules22081356

**Published:** 2017-08-15

**Authors:** Isaiah Cedillo, Dana Chreng, Elyse Engle, Lijian Chen, Andrew K. McPherson, Andrew A. Rodriguez

**Affiliations:** Ionis Pharmaceuticals, Inc., Carlsbad, CA 92010, USA; ICedillo@ionisph.com (I.C.); dchreng@ionisph.com (D.C.); eengle@ionisph.com (E.E.); lchen@ionisph.com (L.C.); amcpherson@ionisph.com (A.K.M.)

**Keywords:** oligonucleotide, GalNAc, conjugate

## Abstract

Antisense oligonucleotides (ASOs) conjugated to triantennary *N*-acetyl galactosamine (GalNAc) ligands represent an emerging approach to antisense therapy. Our current generation of GalNAc-ASO conjugates link the GalNAc to the 5′-terminus of the ASO. The conjugation reaction can be accomplished using solution-phase or solid-phase techniques. Here we show a direct comparison of a solution-phase and a solid-phase conjugation strategy. The solution-phase approach, using amine-pentafluorophenyl (PFP) ester coupling, is higher yielding and gives material of slightly higher purity, but requires several additional unit operations and longer production time. The solid-phase approach, using a protected GalNAc ligand phosphoramidite, is more expedient, but results in lower yield and purity. Both strategies efficiently deliver conjugated material in excellent purity.

## 1. Introduction

Antisense oligonucleotides (ASOs) bind complementary mRNA and modulate its function to yield a pharmacological response [1,2,3]. Second-generation ASOs are typically 16–20 nucleotides in length connected by phosphorothioate linkages and contain a DNA nucleotide “gap” subtended by 2′-*O*-methoxyethyl (MOE) modified RNA “wings”. Kynamro, a second generation ASO targeting Apolipoprotein B-100 mRNA, was recently approved by the FDA for the treatment of homozygous familial hypercholesterolemia [4]. There are more than 100 ASOs advancing in the clinic for a variety of indications [5], many of which target mRNA expressed primarily in the hepatocytes in the liver. Relatively recently, conjugation of ASOs to triantennary *N*-acetyl galactosamine (GalNAc) ligands has been shown to improve potency in hepatocytes [6,7]. GalNAc conjugation on both the 3′ and 5′-termini of the oligonucleotide has been evaluated with the former having slightly enhanced potency in cells and in animals [8]. 

**Figure 1:**
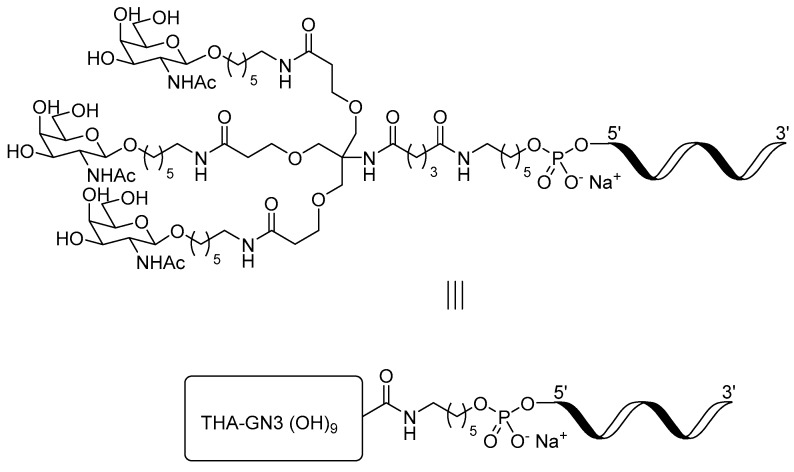
Structure of the GalNAc-ASO conjugate.

The structure of our optimized 5′-GalNAc-ASO conjugate is shown in Figure 1. In the structure, the trishexylamino (THA) GalNAc ligand is connected to the ASO 5′-terminus through an aminohexanol linker. The linker is attached to the ligand via an amide bond and to the ASO via a phosphate diester. Synthetic routes to the GalNAc-ASO conjugate differ in the method and order of formation of both the amide and phosphate diester links.

ASOs are commonly synthesized on a solid support using the phosphoramidite method [9]. The method, which has been highly optimized over the years, involves assembly of the ASO one nucleotide at a time on the support using protected nucleoside phosphoramidite building blocks. The oligonucleotide is typically synthesized in the 3′ ➔ 5′ direction. Numerous synthetic methods have been developed to conjugate molecules to ASOs [10], with conjugation approaches generally falling within two categories: solution-phase and solid-phase. We evaluated each type to synthesize our conjugate structure and report the results herein.

**Figure 2:**
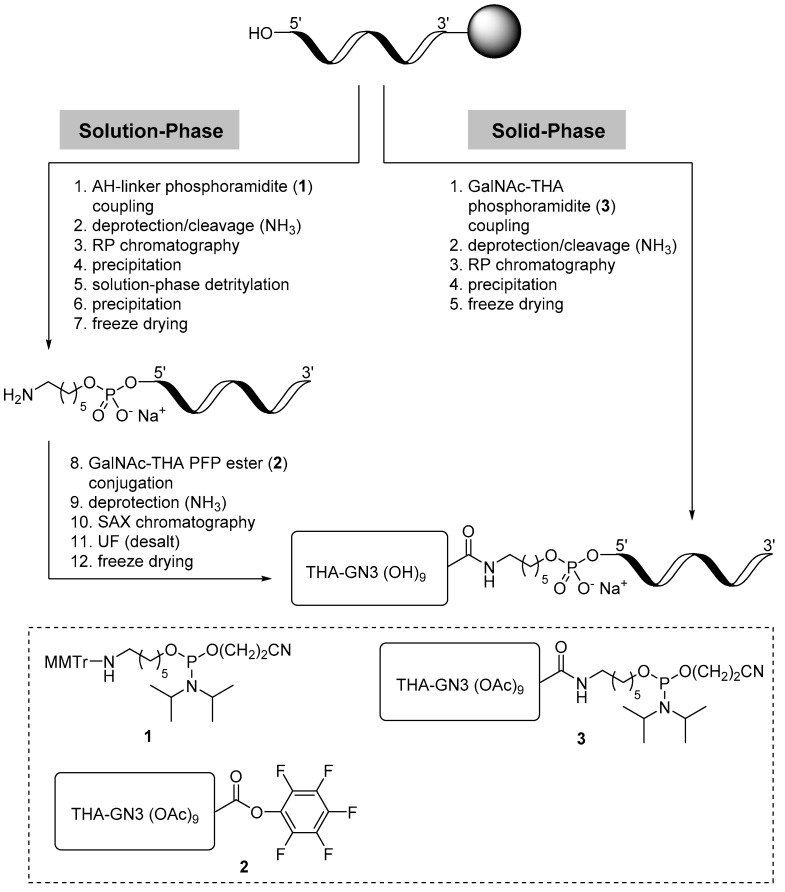
Solution-phase and solid-phase conjugation approaches.

Our initial synthesis route (Figure 2) is an example of a solution-phase approach, consisting of solid-phase synthesis and purification of a 5′-aminohexyl modified ASO, followed by solution-phase conjugation with THA-GalNAc glutarate activated as the pentafluorophenyl (PFP) ester [8]. There are several advantages to this synthetic route. First, the aminohexyl-ASO intermediate can be synthesized, purified, and isolated in high yield and purity using standard oligonucleotide manufacturing techniques. Second, the conjugation reaction is reliable, efficient, and selective. Third, the protocol is scalable, having been successfully used to produce multi-kilogram quantities of conjugated drug substance. The solution-phase strategy, however, is lengthy, involving numerous unit operations, and notably two purification steps. We, therefore, became interested in developing alternative approaches.

We developed an alternative solid-phase conjugation strategy consisting of the coupling of THA-GalNAc-aminohexyl phosphoramidite to support-bound ASO (Figure 2). The process requires fewer unit operations and is considerably shorter than the solution-phase approach. Since there is no trityl-protecting group, a purification protocol capable of discriminating GalNAc-conjugated from unconjugated ASOs was developed.

To evaluate the differences between the solution-phase and solid-phase syntheses outlined above, a head-to-head comparison was conducted. Each process was carried out at 1.1 mmol scale using the same 5-10-5 MOE deoxy gapmer oligonucleotide. The two routes were compared with respect to equivalents of GalNAc-THA required, conjugation efficiency, and purity. The results of the study are presented herein.

## 2. Results and Discussion

### 2.1. THA-GalNAc Phosphoramidite Coupling Optimization

**Scheme 1:**
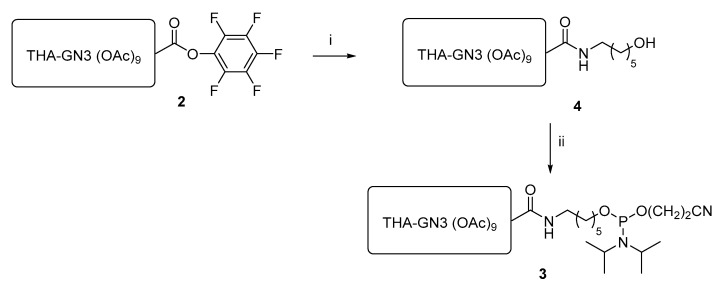
Synthetic protocol for THA-GalNAc phosphoramidite **3**. Reagents and conditions: (**i**) 6-amino-1-hexanol, NEt_3_, THF, 73%; and (**ii**) P(N*^i^*Pr_2_)_2_-O(CH_2_)_2_CN, ETT, CH_2_Cl_2_, 75%.

A considerable amount of optimization was necessary for the GalNAc phosphoramidite conjugation. First, a reliable synthesis of the phosphoramidite itself was developed, starting from PFP ester **2** (Scheme 1). Coupling of **2** with 6-amino-1-hexanol proceeded smoothly with triethylamine in THF to form alcohol **4**. GalNAc-THA phosphoramidite **3** was synthesized with 2-cyanoethyl *N*,*N*,*N’*,*N’*-tetraisopropyl phosphorodiamidite and 5-(ethylthio)-1*H*-tetrazole (ETT) in dichloromethane. Due to the large molecular weight and hydrophilicity of alcohol **4**, it was critical that it be rigorously dried prior to reaction to avoid substantial hydrolysis impurities in the product. Drying was accomplished by multiple azeotropic distillations from dichloromethane.

**Figure 3:**
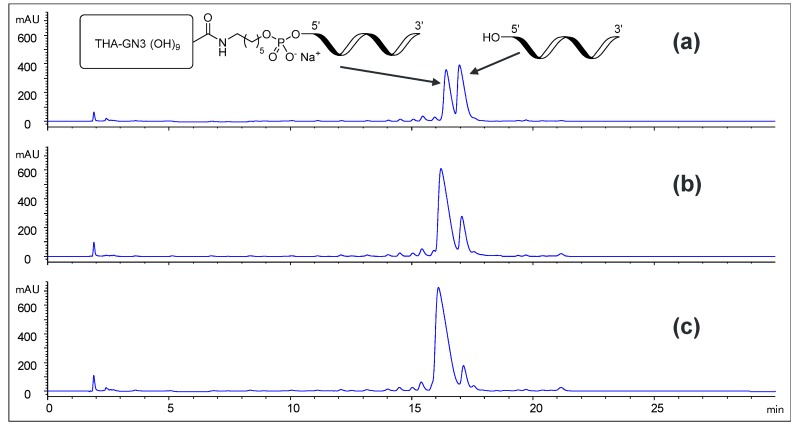
IP-HPLC-UV chromatograms for coupling equivalent study: (**a**) 1.75 equivalents of THA-GalNAc phosphoramidite, (**b**) 3.50 equivalents, and (**c**) 5.25 equivalents.

With phosphoramidite **3** in hand, coupling equivalents were investigated. Our standard nucleotide phosphoramidite coupling protocol was applied three times sequentially to support-bound oligonucleotide, and coupling efficiency was assessed after each cycle. The coupling cycle consists of co-delivery of 1.75 equivalents of 0.20 M THA-GalNAc phosphoramidite solution in acetonitrile and 1.0 M 4,5-dicyanoimidazole (DCI), 0.1 M N-methylimidazole (NMI) in acetonitrile in a 1:1 flow ratio over the course of 1.8 minutes, recirculation through the column for 5 min, oxidation with 0.05 M iodine in 9:1 pyridine:water (*v*/*v*), and washing with acetonitrile. A small sample of support-bound oligonucleotide was taken from the bottom of the synthesis column between each coupling cycle. Ion pair HPLC with ultraviolet detection (IP-HPLC-UV) analysis of the material cleaved from each support sample (Figure 3 and Table 1) indicates that reasonably high coupling efficiency of THA-GalNAc phosphoramidite can be achieved with 5.25 equivalents. Assay of the final bulk sample from this experiment demonstrates an overall UV-pure yield of conjugated oligonucleotide of 58.9%, which is typical for second-generation oligonucleotides. 

Although the overall yield of conjugated oligonucleotide was satisfactory, the large excess of THA-GalNAc phosphoramidite was less than ideal. To address this issue, additional experiments were carried out using extended contact times. Increasing the recirculation time from 5.0 to 15.0 min using 1.75 equivalents increased the coupling efficiency from 46% to 80% (Table 1, entry 1 vs. Table 2, entry 2). Increasing the equivalents to 2.88 and the recirculation time to 30.0 min gave high coupling efficiency and overall yield. The total reagent volume for 2.88 equivalents was approximately equal to the recirculation loop volume, which allowed the entire amount of coupling reagent to remain inside the loop during recirculation.

The impacts of THA-GalNAc coupling temperature, recirculation time, and delivery time were evaluated. The coupling temperature was adjusted using a small shell-and-tube heat exchanger coupled to a recirculating heater/chiller positioned upstream of the synthesis column. Increasing the temperature set point from 21 °C to 45 °C led to a small increase in coupling efficiency to 91% (Table 3, entry 3). Increasing the coupling recirculation time from 30 min to 180 min. resulted in a 94% coupling efficiency (Table 3, entry 4). Increasing coupling delivery time from 1.8 to 8.0 min did not provide any benefits (Table 3, entry 5). Conditions in Table 3, entry 4 resulted in the highest coupling efficiency with 1.75 equivalents of THA-GalNAc phosphoramidite.

### 2.2. Reversed-Phase Chromatography of GalNAc-Conjugated Oligonucleotide

**Figure 4:**
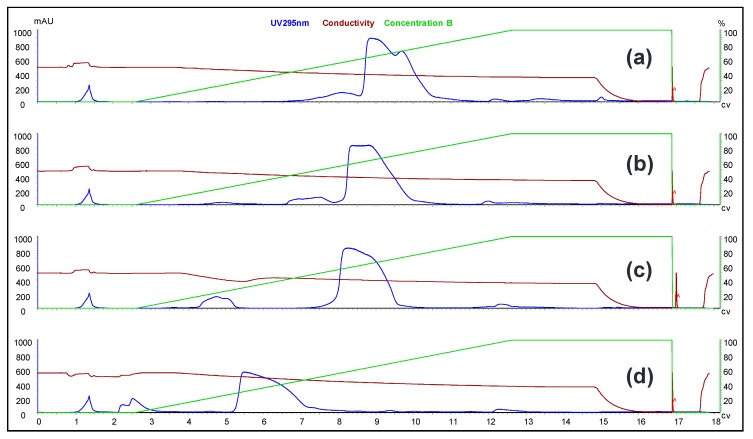
RP-HPLC purification of crude GalNAc-conjugated oligonucleotide. Mobile Phase A is 200 mM NaOAc in 20% MeOH. Mobile Phase B is 200 mM NaOAc in 60% MeOH. Both mobile phases adjusted to: (**a**) pH 7, (**b**) pH 10, (**c**) pH 11, and (**d**) pH 12.

**Figure 5:**
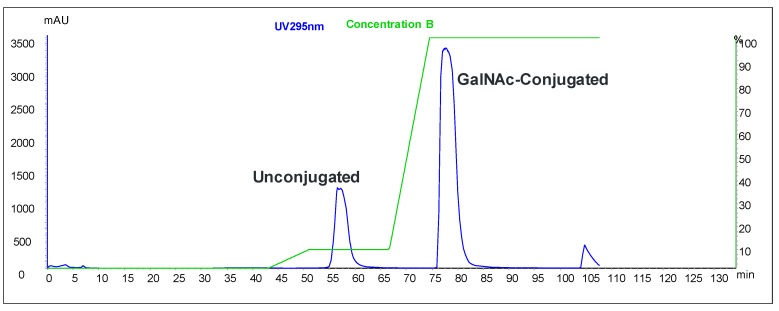
RP-HPLC purification of crude GalNAc-conjugated oligonucleotide with a step gradient. Mobile Phase A is 200 mM NaOAc in 20% MeOH; Mobile Phase B is 200 mM NaOAc in 60% MeOH.

A linear gradient of our standard RP-HPLC buffers (Figure 4a) resulted in only minimal separation of GalNAc-conjugated from unconjugated oligonucleotide. Adjusting the pH of the mobile phases to more basic levels (Figure 4b–d), however, decreased retention times and significantly improved separation. Based on this experiment, a step elution method was developed in which buffer preparation was simplified by adding 20 mM sodium hydroxide in place of a precise buffer pH adjustment, as conducted previously. Complete separation of GalNAc-conjugated from unconjugated oligonucleotide with the new method is demonstrated in Figure 5. Replicate runs (*n* = 11) and fraction analysis demonstrated an average product recovery of 93.2% with an IP-HPLC-UV purity of 97.0%.

### 2.3. Comparison of Solution-Phase and Solid-Phase Conjugation

The solution-phase and solid-phase (phosphoramidite) GalNAc conjugation approaches were directly compared. Support-bound, protected oligonucleotide was prepared by solid-phase synthesis at the 2.2 mmol scale and split into two 1.1 mmol samples. The portion to be used for the solution-phase test was coupled with AHL phosphoramidite, then deprotected and cleaved from the solid support. The crude material was purified by RP-HPLC. The product fraction was precipitated from EtOH, reconstituted in deionized water, detritylated, precipitated a second time, and reconstituted a second time in deionized water. The resulting solution was lyophilized to afford solid aminohexyl-ASO material.

A modified version of the recently published solution-phase conjugation procedure [8] was used to conjugate THA-GalNAc PFP ester to the aminohexyl-ASO. In the current procedure, a solution of 2.6 equivalents of PFP ester **2** in acetonitrile was added to aminohexyl-ASO in 0.06 M sodium tetraborate at pH 9.3 and stirred at room temperature for 3 h (the original procedure used 3.0 equivalents of PFP ester **2**, DMSO instead of acetonitrile, and 0.1 M sodium tetraborate buffer at pH 8.5). It should be noted that the 2.6 equivalents, which is relative to purified aminohexyl-ASO, corresponds to ~1.8 equivalents relative to the starting synthesis scale. Concentrated aqueous ammonia was added and stirred for 24 h. The crude material was purified by SAX chromatography, and desalted by ultrafiltration. The conjugation efficiency was >99%, and the overall yield of the process was estimated to be 58%. HPLC purity of the final product was 97.7%.

The remaining 1.1-mmol portion of support-bound, protected oligonucleotide was coupled to THA-GalNAc phosphoramidite using the conditions from Table 3, entry 4. After completion of synthesis, the oligonucleotide-bound support was dried under vacuum and cleaved from the support with concentrated aqueous ammonia at 55 °C for 15 h. A coupling efficiency of 90% was achieved. The crude solution was purified by RP-HPLC using the step gradient shown in Figure 5. The product fraction was precipitated from EtOH and reconstituted in deionized water. 

**Figure 6:**
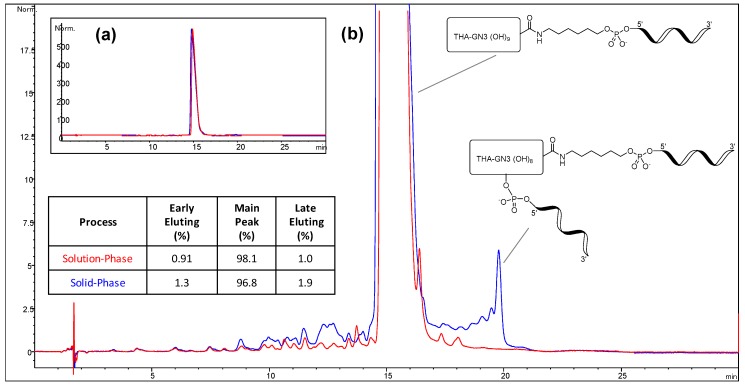
IP-HPLC-UV chromatograms for isolated GalNAc-conjugated ASOs from the solution-phase process (red) and the solid-phase process (blue). (**a**) Full-view chromatograms; and (**b**) zoomed-view chromatograms.

Samples of isolated GalNAc-conjugated oligonucleotide from each process were analyzed by IP-HPLC-UV-MS. A comparison of UV chromatograms is shown in Figure 6. The UV purities of both materials were comparable (98.1% UV purity from the solution-phase approach vs. 96.8% from the solid-phase approach). There was a distinct late eluting peak accounting for ~1% of the UV signal in the sample made from the solid-phase process that was not present in the sample made from the solution-phase process. MS analysis clearly indicated an ion with *m*/*z* = 2634.8 Da. Based on its mass and retention time, the species likely corresponds to the branchmer shown in Figure 6 in the −6 charge state (theoretical most abundant mass = 15,814.1 Da, *m*/*z* = 2634.7 Da in the −6 charge state). The branchmer impurity is likely the result of a bis-phosphoramidite impurity present in the starting material **3**. Studies are ongoing to control the bis-phosphoramidite impurity in the phosphoramidite starting material. The key will be to limit acetyl ester hydrolysis in the phosphoramidite precursor. However, a preliminary purification of the oligonucleotide material by SAX indicates that complete rejection of the branchmer impurity is possible (data not shown).

**Figure 7:**
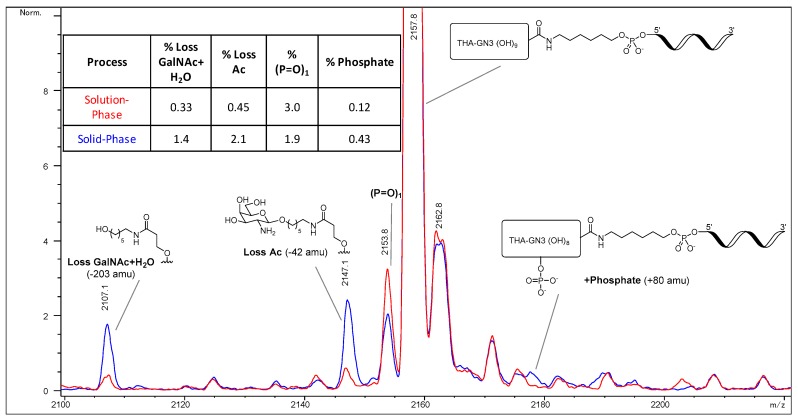
Overlay of average mass spectrum under main UV peaks from the solution-phase process (red) and the solid-phase process (blue).

The average mass spectra under each main peak were examined (Figure 7). The spectra were very similar except for the amounts of four impurities. Material made from the solid-phase method contained significantly higher levels of GalNAc hydrolysis and *N*-acetate hydrolysis impurities, as well as a small increase in a phosphitylated impurity. Material made from the solution-phase method contained ~50% higher (P = O)_1_ impurity. It is not known why GalNAc hydrolysis is more prevalent in the solid-phase approach, but it is likely an acid-catalyzed phenomenon [11]. It is suspected that the hydrolysis arises from trace dichloroacetic acid present on the solid-support at the start of coupling. The increased *N*-acetate hydrolysis in the material made from the solid-phase method arises from heated ammonia treatment during oligonucleotide cleavage. The impurity may be controlled, to some extent, by minimizing ammonia exposure. The slight increase in the phosphate impurity (+80 amu) in the sample made from the solid-phase method is probably related to the branchmer impurity; failure of one of the phosphoramidite groups on the bis-phosphoramidite to couple is expected to result in this impurity. The increased (P = O)_1_ impurity in the material made from the solution-phase method is of unclear origin, but it appears to arise during or after conjugation. We suspect the cause is ammonolysis in the presence of acetonitrile, which has been observed to lead to measurable desulfurization at 55 °C (unpublished results) with other compounds. It is possible that, even at room temperature, the large amount of acetonitrile present in the solution-phase conjugation reaction during ammonolysis increases the impurity to the observed extent. Another possibility is simple phosphorothioate hydrolysis during the basic conditions of conjugation (pH of conjugation solution is ~9.3).

## 3. Materials and Methods 

### 3.1. Materials

Low water acetonitrile from BDH Chemicals (purchased through VWR, Radnor, PA, USA), deblocking reagent from EMD Millipore (Billerica, MA, USA), oxidation solution from EMD Millipore, xanthane hydride from TCI (Tokyo, Japan), pyridine from Alfa Aesar (Tewksbury, MA, USA), acetic anhydride from Avantor Peformance Chemicals (Center Valley, PA, USA), triethylamine from Burdick and Jackson (Morristown, NJ, USA), and toluene from BDH were all purchased via VWR (Radnor, PA, USA). Molecular sieve packets were purchased from Prime Synthesis (Aston, PA, USA). NittoPhaseHL UnyLinker solid support was purchased from Kinovate Life Sciences (Oceanside, CA, USA).

### 3.2. THA-GalNAc Aminohexyl Alcohol ***4***

THA-GalNAc PFP ester **2** (150 g, 78.8 mmol) was dissolved in THF (750 mL) with stirring. Triethylamine (27.5 mL, 197 mmol) was added, followed by 6-amino-1-hexanol (9.2 g, 78.8 mmol). The mixture was stirred for 1 h at room temperature and diluted in CH_2_Cl_2_ (500 mL). The solution was washed once with 500 mL 5% NaHSO_4_ solution and twice with 500 mL saturated NaHCO_3_ solution. The organic layer was dried over Na_2_SO_4_, filtered, and concentrated. The crude material was purified by SiO_2_ chromatography with a gradient of 5% MeOH in EtOAc up to 20% MeOH in EtOAc. The product fractions were combined and concentrated to a white solid (111.9 g, 77% yield). 

^1^H-NMR (300 MHz, CD_3_CN) δ 6.99 (t, *J* = 5 Hz, 3H), 6.85 (d, *J* = 9 Hz, 3H), 6.71 (m, 1H), 6.50 (m, 1H), 5.35 (d, *J* = 3 Hz, 3H), 5.26 (dd, *J* = 5, 11 Hz, 3H), 4.67 (d, *J* = 9 Hz, 3H), 4.15 (m, 6H), 3.93 (m, 9H), 3.67 (m, 14H), 3.49 (m, 3H), 3.23 (m, 8H), 2.42 (t, *J* = 6 Hz, 6H), 2.23 (m, 4H), 2.14 (s, 9H), 2.05 (s, 9H), 2.00 (s, 9H), 1.95 (s, 11H), 1.53 (m, 16H), 1.36 (m, 16H); ^13^C-NMR (75 MHz, CD_3_CN) δ 173.1, 173.0, 171.5, 170.5, 170.4, 107.9, 101.1, 77.3, 70.5, 70.2, 69.6, 69.3, 67.5, 66.9, 62.3, 61.5, 59.7, 53.5, 51.3, 39.3, 36.7, 36.3, 35.3, 32.5, 31.9, 29.5, 29.2, 29.0, 26.5, 25.5, 25.3, 24.0, 23.3, 22.7, 22.3, 20.7, 14.1.

### 3.3. THA-GalNAc Phosphoramidite ***3***

THA-GalNAc aminohexyl alcohol **4** (18.1 g, 9.86 mmol) was dissolved in CH_2_Cl_2_ (54 mL) in a round-bottomed flask. The solution was concentrated to dryness by rotary evaporation. The solid was redissolved in CH_2_Cl_2_ (54 mL) and the solution concentrated to dryness two more times. The solid was redissolved in CH_2_Cl_2_ (54 mL) under positive N_2_ pressure. The solution was cooled to 0 °C in an ice bath. With stirring, 2-cyanoethyl-*N*,*N*,*N′*,*N′*-tetraisopropylphosphordiamidite (5.94 g, 19.72 mmol) was added dropwise by syringe. The solution stirred for 5 min. Ethylthiotetrazole (ETT, 1.54 g, 11.83 mmol) was added as a solid. The solids slowly dissolved into a homogeneous solution. The reaction mixture stirred for 3 h at 0 °C. After 3 h, the reaction mixture warmed to room temperature and triethylamine (5.0 mL, 35.85 mmol) was added dropwise. The mixture was concentrated to ~⅓ volume by rotary evaporation and loaded onto a SiO_2_ column (100 g SiO_2_, ~10 cm diameter) that had been prepared with EtOAc containing 1% NEt_3_. The column was flushed with 2 L of 1% NEt_3_ in EtOAc followed by 2 L of 1:3:96 NEt_3_:acetone:THF. The product eluted during the first 1 L of the latter mobile phase mixture. Product fractions were combined and concentrated to a white solid (15.1 g, 75% yield).

^1^H-NMR (300 MHz, CD_3_CN) δ 6.97 (m, 3H), 6.83 (m, 3H), 6.53 (s, 1H), 5.47 (s, 1H), 5.30 (d, *J* = 3 Hz, 3H), 5.05 (dd, *J* = 5, 11 Hz, 3H), 4.55 (d, *J* = 6 Hz, 3H), 4.10 (m, 6H), 3.98 (m, 6H), 3.82 (m, 4H), 3.63 (m, 15H), 3.51 (m, 3H), 3.16 (m, 8H), 2.66 (t, *J* = 6 Hz, 2H), 2.35 (t, *J* = 6 Hz, 6H), 2.16 (m, 4H), 2.12 (s, 9H), 2.01 (s, 9H), 1.96 (p, *J* = 3 Hz, 2H), 1.94 (s, 9H), 1.87 (s, 9H), 1.82 (m, 2H), 1.48 (m, 14H), 1.41 (s, 6H), 1.34 (m, 12H), 1.20 (dd, *J* = 2, 8 Hz, 12H); ^31^P-NMR (121.5 MHz, CD_3_CN) δ 147.05.

### 3.4. Solid-Phase Synthesis 

A total of 2.2 mmol (7.0 g) of NittoPhaseHL UnyLinker solid support loaded at 317 µmol/g was weighed into a plastic weigh boat, slurried in acetonitrile, and transferred to a FineLINE 35 mm column (GE Healthcare, P/N 28946841, Little Chalfont, UK) with the outlet plugged. Additional acetonitrile was added to approximately 10 cm and then the piston was lowered to a height of approximately 7.0 cm. Synthesis was performed using an AKTA OligoPilot 100 Plus synthesizer (GE Healthcare, P/N 18-1136-79) equipped with custom mass flow meters. 

Dichloroacetic acid (10% by volume) in toluene was used for deblocking of the 4,4′-dimethoxytrityl (DMTr) groups from the 5′-hydroxyl group of the nucleotide. 4,5-Dicyanoimidazole (1.0 M) in the presence of *N*-methylimidazole (0.10 M) was used as the activator during the coupling step. During the coupling step, 1.45 equivalents of 0.20 M phosphoramidite solution (2′-deoxy and 2′-*O*-methoxyethyl nucleosides) and a flow ratio of 1:1 (*v*/*v*) of phosphoramidite solution to activator solution was used. Phosphoramidite and activator solutions were prepared using low-water acetonitrile (water content <30 ppm) and were dried further by the addition of molecular sieve packets (Prime Synthesis, P/N SP-MT10). Phosphorothioate linkages were introduced by sulfurization of phosphite triesters with 0.20 M solution of xanthane hydride in pyridine. Phosphate diester linkages were incorporated via oxidation of phosphite triesters using a solution of iodine in pyridine/water (90/10, *v*/*v*). Unreacted hydroxyl groups were acetylated using *N*-methylimidazole/pyridine/acetonitrile (20/30/50, *v*/*v*/*v*) and acetic anhydride/acetonitrile (20/80, *v*/*v*) delivered in a 1:1 (*v*/*v*) flow ratio. At the end of synthesis, the support-bound oligonucleotide was treated with a solution of triethylamine/acetonitrile (1:1, *v*/*v*) to remove acrylonitrile formed by deprotection of the cyanoethyl group from the phosphorothioate triester. Reagent delivery volumes and contact times are detailed in Table 4. Subsequently, the support-bound oligonucleotide was incubated with 160 mL of concentrated aqueous ammonium hydroxide at 55 °C for approximately 15 h to complete the cleavage from the support, elimination of UnyLinker molecules to liberate the 3′-hydroxy group of the oligonucleotide, and deprotection of nucleobase-protecting groups. After allowing the crude mixture to cool to room temperature it was filtered (0.45 µm aPES) and the native support was rinsed with 160 mL of purified water. 

### 3.5. Work-Up and Isolation of Solid-Phase GalNAc-ASO Conjugate

Precipitation of purified oligonucleotide was performed by adding 1.0 part purified oligonucleotide solution to 8.0 or 9.0 parts ethanol by volume in a glass bottle. The precipitation mixture was shaken vigorously by hand and allowed to settle overnight at room temperature. Supernatant was decanted to waste and precipitated oligonucleotide was reconstituted in water. Replicate runs (*n* = 6) demonstrated that measured recovery was 98–100% by IP-HPLC-UV.

### 3.6. Solution-Phase GalNAc-THA PFP Ester Conjugation

The following procedure is based on a previously reported protocol [8]. Aminohexyl-ASO (1.8 g, 0.23 mmol) was dissolved in 0.06 M sodium tetraborate buffer at pH 9.3 (18 mL) and stirred at room temperature. A solution of THA-GalNAc PFP ester **2** (1.137 g, 0.60 mmol, 2.6 eq. vs. aminohexyl-ASO, 1.8 eq. vs. 1.1 mmol solid-phase synthesis scale) dissolved in acetonitrile (6.4 mL) was added to the aminohexyl-ASO solution over about one minute. The mixture was stirred at room temperature for 3.25 h, at which point in-process analysis indicated the reaction had progressed to >99%. Concentrated aqueous ammonia was added (10 mL), and the reaction mixture stirred for 24 h at room temperature. The crude solution was purified by SAX chromatography using SOURCE 30Q media (GE Healthcare) and a gradient of Buffer A (20 mM NaOH in water) and Buffer B (2 M NaCl, 20 mM NaOH in water). Product fractions were combined and desalted by ultrafiltration (Sartorius 5 kD Hydrosart membrane). The conjugation efficiency was >99%, and the overall yield of the process was estimated to be 58%. HPLC purity of the final product was 97.7%. 

### 3.7. IP-HPLC-UV-MS Analysis 

A sample of crude solution was weighed into a centrifuge, vacuum centrifuged to dryness at room temperature, and reconstituted in approximately 1 mL of water. Reconstituted solution was transferred to a 10 mL volumetric flask and brought to volume with water. Purified and isolated samples were prepared by dilution in water. Prepared samples were analyzed on an Agilent 1200 series HPLC (Santa Clara, CA, USA) coupled to an Agilent 6130 Quadrapole mass spectrometer using an XBridge C18 3.5 µm 2.1×150 mm column (Waters P/N 186003023, Milford, MA, USA) using the gradient shown in Table 5. UV detection was set to 260 nm with a 4-nm bandwidth and a reference wavelength of 400 nm with an 80 nm bandwidth. Column temperature was set to 50.0 °C.

## 4. Conclusions

ASOs with 5′-GalNAc conjugation can be synthesized in high yield and purity using two different methods. The solution-phase method [8] consists of conjugation of purified aminohexyl-ASO to THA-GalNAc PFP ester in an aqueous buffer. The solid-phase method consists of coupling of a preformed THA-GalNAc phosphoramidite to the support-bound oligonucleotide. The solid-phase approach required significant optimization to be viable. As a result of the optimization work presented herein, THA-GalNAc phosphoramidite can be coupled consistently in >90% coupling efficiency, and the GalNAc-conjugated crude oligonucleotide can be conveniently purified from unconjugated species using a modified RP-HPLC method.

The two methods were compared at 1.1 mmol scale with the same 5-10-5 MOE-gapmer oligonucleotide and roughly the same molar excess of THA-GalNAc species. Solution-phase conjugation produced material in somewhat higher yield (58% vs. 47%) and UV purity (97.7% vs. 96.6%) than solid-phase conjugation. Most of the UV purity difference was due to a branchmer impurity only present in the sample made from the solid-phase method. On the other hand, the solid-phase conjugation procedure is significantly simpler and faster than the solution-phase procedure, requiring a single purification step and a single lyophilization step. Different levels of specific impurities were observed by MS in samples made from the two methods. Solid-phase conjugation led to more GalNAc hydrolysis (1.4% vs. 0.33%), *N*-acetate hydrolysis (2.1% vs. 0.45%), and phosphate impurities (0.43% vs. 0.12%), but solution-phase conjugation led to more (P = O)_1_ impurity (3.0% vs. 1.9%). 

Current efforts are focused on improving the yield and purity of GalNAc-conjugated ASOs produced from both methods.

## Figures and Tables

**Table 1:** Impact of phosphoramidite equivalents on coupling efficiency.

Entry	Phosphoramidite (Eq.)	Total Time (min)	Efficiency ^1^ (%)
1	1.75	6.8	46
2	3.50	13.6	79
3	5.25	20.4	94

^1^ Coupling efficiency of THA-GalNAc estimated based on UV area of conjugated relative to unconjugated.

**Table 2:** Impact of phosphoramidite equivalents on coupling efficiency.

Entry	Phosphoramidite (Eq.)	Delivery (min)	Recir. Time (min)	Efficiency ^1 ^(%)
1	1.75 × 3 = 5.25	1.8 × 3 = 5.4	5.0 × 3 = 15.0	94
2	1.75	1.8	15.0	80
3	2.88	2.3	30.0	93

^1 ^Coupling efficiency of THA-GalNAc estimated based on UV area of conjugated relative to unconjugated.

**Table 3:** Impact of THA-GalNAc phosphoramidite equivalents and reaction temperature on coupling efficiency.

Entry	Phosphoramidite (Eq.)	Delivery (min)	Recirc. Time (min)	Temp. (°C)	Efficiency ^1 ^(%)
1	1.75	1.8	15.0	19	80
2	1.75	1.8	30.0	21	87
3	1.75	1.8	30.0	45	91
4	1.75	1.8	180.0	21	94
5	1.75	8.0	180.0	19	90

^1 ^Coupling efficiency of THA-GalNAc estimated based on UV area of conjugated relative to unconjugated.

**Table 4:** Reaction parameters for 2.2-mmol scale synthesis on the AKTA OligoPilot 100 synthesizer.

Step	Cycle	Delivery Volume (mL)	Delivery Time (min)
Detritylation	1–16	140.08	2.63
	17–20	168.32	3.16
Coupling (MOE, Deoxy)	1–20	30.98	1.62 + 3.25 *
Sulfurization	1–20	71.10	2.96
Oxidation	21	87.88	3.52
Capping	12–20	71.1417.62	2.98 + 5.00 *0.77
Phosphorus Deprotection	NA	102.16	1.98 + 15.00 *

* reagents recirculated back through the column

**Table 5:** Gradient parameters for HPLC analysis of crude oligonucleotide. Data acquisition time is 30 min with a 10 min post time for column equilibration. Buffer A is 5 mM tributylammonium, 1 µM EDTA in 10% acetonitrile. Buffer B is 5 mM tributylammonium acetate, 1 µm EDTA in 80% acetonitrile. Flow rate is 0.25 mL/min.

Time (min)	Buffer A (%)	Buffer B (%)
0	55	45
22	20	80
25	20	80
26	55	45
